# Intra-articular injection of hyaluronic acid for the reduction in joint adhesion formation in a rabbit model of knee injury

**DOI:** 10.1007/s00167-013-2547-3

**Published:** 2013-06-06

**Authors:** Min Wang, Chao Liu, Wei Xiao

**Affiliations:** Department of Orthopaedics, Xinqiao Hospital, Third Military Medical University, No. 2 Xinqiao Street, Shapingba District, Chongqing, 400037 China

**Keywords:** Intra-articular injection, Hyaluronic acid, Joint adhesion, Knee

## Abstract

**Purpose:**

Our purpose was to evaluate the effectiveness of intra-articular injections of hyaluronic acid (HA) into immobilized joints for reducing rigidity and formation of joint adhesions following surgery and prolonged joint immobilization.

**Methods:**

Twenty-four New Zealand white rabbits were randomly divided into experimental (*n* = 12) and control groups (*n* = 12). A model of knee injury was created in the right hind leg, and external plaster fixation was performed for 8 weeks. The experimental and control groups received weekly intra-articular injections of 0.3 mL HA solution or normal saline, respectively, in the knee joint. The degree of adhesions, range of motion (ROM), and collagen content of the synovium of the knee joint were observed after 8 weeks.

**Results:**

At the end of 8 weeks, the experimental compared with control group had significantly higher mean ROM (70.3° ± 11.1° vs. 54.6° ± 11.2°, respectively; *P* = 0.002) and mean adhesion score. The experimental group compared with the control group had significantly lower mean adhesion score (2.2 ± 0.9 vs. 3.1 ± 0.7, respectively; *P* = 0.012) and collagen content (32.4 ± 4.7 vs. 39.0 ± 4.2 μg/mg, *P* = 0.001).

**Conclusions:**

In a rabbit model of knee injury, intra-articular injection of HA decreased adhesion formation and collagen content and increased ROM after prolonged immobilization. These results indicate that HA may be clinically useful to prevent adhesions and improve joint mobility in patients who require joint immobilization for up to 8 weeks.

## Introduction

Joint adhesion formation begins with an inflammatory response and can be regarded as tissue fibrosis [6, 10, 13, 18]. Postoperative adhesions, which can be aggravated by prolonged joint immobilization, can result in loss of motion, pain, and stiffness and can significantly affect the activities of daily life [6]. Long-term joint adhesions often cause further fibrosis and contracture of the joint capsule and ligaments, resulting in ankylosis which causes yet further deterioration of the joint and additional stiffness [6]. Once ankylosis occurs, restoration of normal joint motion and function is difficult [6]. Types of patients who may require joint immobilization for extended periods of time include those receiving arthroscopic anterior cruciate ligament (ACL) or posterior cruciate ligament (PCL) reconstruction, cartilage tissue engineering, joint debridement after serious trauma, and those with intra-articular or periarticular fractures.

Hyaluronic acid (HA) is a high-molecular-mass straight-chain polysaccharide that is widely distributed in human tissues [9]. Its irregular coiled state and hydrodynamic characteristics in solution endow it with high degrees of viscoelasticity, plasticity, and permeability [9]. Intra-articular injection of HA is recognized as an effective method to reduce the friction of articular cartilage and is widely used in the treatment and prevention of osteoarthritis [5, 15, 23, 25, 26]. A number of studies have suggested that the use of HA can prevent postoperative adhesions in the rotator cuff [20], knee [3, 12], temporomandibular joint [19], flexor tendons [21], and abdomen [2]. A recent systematic review by Harris et al. [11] found that injection of HA into the glenohumeral joint is a safe and effective treatment for the management of adhesive capsulitis.

HA has previously been reported to reduce pain and improve osteoarthritis after immobilization of 4–5 weeks [9]. However, some patients who receive joint surgery will have the joint immobilized by a cast for about 8 weeks [24]. The purpose of this study was to investigate the effects of intra-articular injection of HA for the prevention of knee ankylosis in a rabbit model of knee injury in which the joint was immobilized for 8 weeks. We hypothesized that the application of hyaluronic acid for 8 weeks would reduce joint adhesions and collagen content and increase the range of motion as compared with controls.

## Materials and methods

### Construction of the rabbit model of traumatic knee adhesions

Twenty-four New Zealand white rabbits were randomly divided into an experimental group and a control group (*n* = 12 in each group). The intervention was performed on the right knee of the rabbits. The rabbits were anesthetized through intravenous injection of 3 % pentobarbitol (1 mL/kg) via the marginal ear vein, and the model was constructed as described by Fukui et al. [7]. In brief, the hair around the knee joint of the right hind leg was removed using small electric clippers, and the skin was disinfected with povidone iodine. Under sterile conditions, an incision of approximately 3 cm was made lateral to the patella in the right knee on the inner side. The joint capsule was cut open, and the patella was turned outwards. A scalpel blade was used to scratch the synovial membrane on the anterior wall of the suprapatellar bursa, and the scalpel handle was used to push away the attachment between the posterior wall of the suprapatellar bursa and the anterior femur to partially resect the posterior wall of the suprapatellar bursa and infrapatellar fat pad. A sample of tissue was obtained for testing of the baseline collagen content. Haemostasis was performed, and the joint capsule and skin were sutured. Joint puncture was performed to aspirate the intra-articular haematocele, and then HA was injected into the joint cavity.

The rabbits in the experimental and control groups received a 0.3 mL (3 mg) intra-articular injection of HA (Sodium Hyaluronate Injection ARTZ^®^ Dispo 25 mg/mL, Seikagaku Corp., Japan) or 0.3 mL normal saline, respectively, in the knee joint weekly for 8 weeks. A long-leg circular cast was applied after surgery, and rabbits underwent postoperative external plaster fixation of the right hind limb for 8 weeks with the knee joint extended. A window was opened in the plaster in front of the knee joint so that HA or saline could be injected. The concentration of HA was based on a prior study [23, 28]. No interventions were performed on the contralateral limb. Rabbits were housed in individual cages after surgery and received intramuscular injection of gentamicin 20,000 units twice per day for 3 days. The cast was removed after 8 weeks, and measurement of range of motion (ROM) of the operated limb was performed. The rabbits were then anesthetized for general observation of the intra-articular adhesions, and synovial tissue on the anterior wall of the suprapatellar bursa was collected to determine the total collagen content.

The ROM of the knee was measured using a plastic goniometer with the rabbit lying on its side at room temperature (22 °C). The goniometer used three points (proximal femur, distal tibia, and lateral femoral condyle) to measure the knee joint angle. The fulcrum was centred over the lateral knee joint space, by palpating the soft-tissue depression just posterior to the lateral tibial plateau. This was the pivot point from which the angle between the tibia and femur was measured. One arm of the goniometer was placed parallel to the line between the greater trochanter and the knee joint, and the other arm was placed parallel to the longitudinal axis of the lower leg and over the ankle at mid-width. Torque of approximately 750–850 g was applied manually to the knee joint during the knee extension measurement, and the distance between the knee joint and the finger placement on the animal’s foot was about 9 cm. An angular velocity of approximately 3°/s was standard. Only one researcher performed the ROM measurement, and he was blinded to group assignment. Three measurements were performed, and the average value was calculated.

Observation of articular adhesions was assessed using Rothkopf’s visual scoring system of bone and joint capsule adhesions [22] by an experienced pathologist who specialized in joint disease and who was blinded to the study. The degree of adhesion formation was scored: 0, no adhesions; 1, weak, mild adhesions in which the adhesion membrane was thin and could be stripped by minimal manual traction; 2, moderate adhesions which could be removed by manual traction; and 3, dense adhesion layer that could only be removed surgically.

Synovial tissue samples were collected at baseline and after 8 weeks and were stored in a freezer at −20 °C. A 3 mg sample was used for measuring hydroxyproline content using a hydroxyproline kit (Santa Cruz Biotechnology, Santa Cruz, CA, USA) per the manufacturer’s instructions. The total collagen content was calculated based on the fact that collagen contains 13.4 % hydroxyproline, and results were expressed as μg collagen/mg tissue. This study was approved by the institutional review board of Xinqiao Hospital, Clinical Medical Research Center, the Third Military Medical University, Chongqing, China (approval number: xq2011-022), and all animals were treated according to standard guidelines for the ethical treatment and use of animals in research.

### Statistical analysis

Eight rabbits, which were not included in this study, were evaluated in a pilot study. It was supposed that the variances of collagen level in the two groups were equal. The results of pilot study showed that the observed variance of the two groups, $S_{1}^{2} = S_{2}^{2} = 15$, and the mean difference of collagen, *x*_1_ − *x*_2_, was 3.3. Thus, the sample size in each group should be at least 11 according to the equation $N = \frac{{t_{\alpha }^{2} \cdot (S_{1}^{2} + S_{2}^{2} )}}{{(x_{1} - x_{2} )^{2} }}$, where *α* = 0.05 was the significance level. Based on these calculations, we used 24 rabbits, 12 in each group.

Results were presented as mean ± SD, and the differences in ROM, adhesions, and collagen content were assessed by independent two sample *t* test. The data were normally distributed, and all statistic assessments were evaluated at a two-sided alpha level of 0.05. Statistical analyses were performed using SAS 9.2 statistics software (SAS Institute Inc., Cary, NC, USA).

## Results

The baseline ROM and collagen content of the knee joint were not different between the two groups (Table 1). All procedures were performed without complications, and all animals remained in good health throughout the 8-week experimental period. No wound infections, self-mutilation, or deaths occurred. Various degrees of swelling of the ipsilateral foot occurred in some animals (*n* = 4 in the control and *n* = 5 in the experimental group) due to tight bandaging, and in severe cases, the distal plaster was partially loosened. The plaster was repaired or replaced when damaged to ensure joint immobility at all times.

**Table 1:** Baseline data

	Control group	Experimental group	*P* value
Range of motion (°)	170.0 ± 2.9	168.8 ± 2.2	n.s.
Collagen content (μg/mg tissue)	29.8 ± 2.2	30.0 ± 2.1	n.s.

Data presented as mean ± SD and difference between groups was assessed by independent two sample *t* test

*n.s.* non-significant

Table 2 summarizes the effects of HA injection on ROM, formation of joint adhesion, and collagen content following 8 weeks of treatment. The experimental group had significantly higher mean ROM (70.3° ± 11.1° vs. 54.6° ± 11.2°, respectively, *P* = 0.002), significantly lower mean joint adhesion score (2.2 ± 0.9 vs. 3.1 ± 0.7, respectively, *P* = 0.012), and significantly lower mean collagen content (32.4 ± 4.7 vs. 39.0 ± 4.2 μg/mg, respectively, *P* = 0.001).

**Table 2:** Summary of joint assessments

	Control group	Experimental group	*P* value
Range of motion (°)	54.6 ± 11.2	70.3 ± 11.1	0.002
Rothkopf’s score	3.1 ± 0.7	2.2 ± 0.9	0.012
Collagen content (μg/mg tissue)	39.0 ± 4.2	32.4 ± 4.7	0.001

## Discussion

The most important finding of the present study was that intra-articular administration of HA during 8 weeks of knee immobilization significantly reduced the degree of joint adhesion formation, lowered the collagen content, and increased the ROM. Some joint surgeries require immobilization for up to 8 weeks, and our findings indicate a possible clinical benefit of injecting HA into joints immobilized for up to 8 weeks. Joint adhesions often appear after 4 weeks [6] of immobilization, hence the 8-week duration of this study allowed for a more critical analysis of the effect of HA on preventing joint adhesions.

HA is a linear polysaccharide that is composed of a disaccharide unit, *N*-acetyl glucosamine, and glucuronic acid [16]. It is secreted by synovial B cells and mononuclear phagocytes in the joint, and it is an important component of synovial fluid and the articular cartilage matrix, exerting a number of physiological functions including lubrication, shock absorption, infection resistance, and participation in cartilage repair [5]. HA inhibits the activity of fibroblasts and inhibits the local inflammatory response [23]. HA also reduces pain, which is conducive to early rehabilitation and exercise, by blocking activation of pain neurons [27].

Currently, there are a number of pharmacological methods for preventing and mitigating joint adhesions including intra-articular injection of corticosteroids, decorin, chitosan, mitomycin C, and inhibitors of transforming growth factor (TGF)-β [4, 7, 8, 17, 28]. However, none of these methods have proven to be ideal.

Studies have shown the benefits of replenishment of HA including significant decreases in inflammation of synovial tissue, increases in the viscosity of the synovial fluid and its lubricating function, increases ROM, promotion of healing and regeneration of articular cartilage, and promotion of HA synthesis [5, 26]. HA injection resulted in faster recovery compared with no HA treatment in forward flexion after arthroscopic rotator cuff surgery [20] and reduced pain following arthroscopic lysis and lavage in patients with Wilkes stage III and IV disease [19]. Hyaloglide, a hyaluron-based gel, resulted in better recovery of finger motion after tenolysis of flexor tendons in zone II [21] and reduced joint adhesion [3]. A recent systematic review [11] found that injection of HA is a safe and effective in managing adhesive capsulitis of the shoulder. A meta-analysis of intra-articular injection of HA for the treatment of osteoarthritis found that compared with placebo HA resulted in measurable beneficial effects on joint function by 4 weeks, reached peak effectiveness at 8 weeks, and exerted a residual effect up to 24 weeks [1].

There are certain limitations to this study that should be considered when interpreting the findings. The study had a small sample size and did not assess if HA had a dose effect or resulted in side effects. This study used a cast for immobilizing the knee, but it is possible that an external fixator may have been more effective at complete immobilization of the joints. Changes in weight, the nutritional status of the lower limb, and other parameters that may have affected joint activity were not assessed. Additionally, test–retest reliability measurements were not performed. Lastly, this study used a rabbit model, and the results may not be applicable to human joints; however, prior study has shown that changes in collagen and other factors in the rabbit posterior knee immobilized for 8 weeks were similar to findings in the anterior joint capsule of humans with chronic posttraumatic elbow joint contractures [14].

## Conclusions

In this rabbit model of knee injury, intra-articular injection of HA decreased adhesion formation, reduced collagen content, and increase ROM after prolonged immobilization. These results indicate that HA may be clinically useful to prevent and adhesions improve joint mobility in patients who require joint immobilization for up to 8 weeks.

## References

[1] Bannuru RR, Natov NS, Dasi UR, Schmid CH, McAlindon TE (2011). Therapeutic trajectory following intra-articular hyaluronic acid injection in knee osteoarthritis–meta-analysis. Osteoarthritis Cartilage.

[2] Belluco C, Meggiolaro F, Pressato D, Pavesio A, Bigon E, Dona M (2001). Prevention of postsurgical adhesions with an autocrosslinked hyaluronan derivative gel. J Surg Res.

[3] Brunelli G, Longinotti C, Bertazzo C, Pavesio A, Pressato D (2005). Adhesion reduction after knee surgery in a rabbit model by Hyaloglide, a hyaluronan derivative gel. J Orthop Res.

[4] Chen B, Guo B, Zhoa Z (2002). Effects of chitosan membrane placement in preventing postoperative adhesion of knee joint. Chin J Orthop.

[5] Curran MP (2010). Hyaluronic acid (Supartz^®^): a review of its use in osteoarthritis of the knee. Drugs Aging.

[6] Eakin CL (2001). Knee arthrofibrosis: prevention and management of a potentially devastating condition. Phys Sportsmed.

[7] Fukui N, Fukuda A, Kojima K, Nakajima K, Oda H, Nakamura K (2001). Suppression of fibrous adhesion by proteoglycan decorin. J Orthop Res.

[8] Fukui N, Tashiro T, Hiraoka H, Oda H, Nakamura K (2000). Adhesion formation can be reduced by the suppression of transforming growth factor-beta1 activity. J Orthop Res.

[9] Gaffney J, Matou-Nasri S, Grau-Olivares M, Slevin M (2010). Therapeutic applications of hyaluronan. Mol BioSyst.

[10] Griffin M, Hindocha S, Jordan D, Saleh M, Khan W (2012). An overview of the management of flexor tendon injuries. Open Orthop J.

[11] Harris JD, Griesser MJ, Copelan A, Jones GL (2011). Treatment of adhesive capsulitis with intra-articular hyaluronate: A systematic review. Int J Shoulder Surg.

[12] Hayashi M, Sekiya H, Takatoku K, Kariya Y, Hoshino Y (2004). Experimental model of knee contracture in extension: its prevention using a sheet made from hyaluronic acid and carboxymethylcellulose. Knee Surg Sports Traumatol Arthrosc.

[13] Hsu C, Chang J (2004). Clinical implications of growth factors in flexor tendon wound healing. J Hand Surg Am.

[14] Huang CB, Ye YG, Xiang XB, Xiong P (2010). Effects of tendon and collateral relaxing formula on the local tissue in rabbit model of traumatic knee adhesion. J Tradit Chin Med.

[15] Hulmes DJ, Marsden ME, Strachan RK, Harvey RE, McInnes N, Gardner DL (2004). Intra-articular hyaluronate in experimental rabbit osteoarthritis can prevent changes in cartilage proteoglycan content. Osteoarthritis Cartilage.

[16] Isayeva I, Sarkar Das S, Chang A, Defoe J, Luu HM, Vorvolakos K (2010). pH effect on the synthesis, shear properties, and homogeneity of iron-crosslinked hyaluronic acid-based gel/adhesion barrier. J Biomed Mater Res B Appl Biomater.

[17] Kocaoglu B, Akgun U, Nalbantoglu U, Poyanli O, Karahan M (2011). Adhesion reduction after knee surgery in a rat model by mitomycin C. Knee Surg Sports Traumatol Arthrosc.

[18] Molloy T, Wang Y, Murrell G (2003). The roles of growth factors in tendon and ligament healing. Sports Med.

[19] Morey-Mas MA, Caubet-Biayna J, Varela-Sende L, Iriarte-Ortabe JI (2010). Sodium hyaluronate improves outcomes after arthroscopic lysis and lavage in patients with Wilkes stage III and IV disease. J Oral Maxillofac Surg.

[20] Oh CH, Oh JH, Kim SH, Cho JH, Yoon JP, Kim JY (2011). Effectiveness of subacromial anti-adhesive agent injection after arthroscopic rotator cuff repair: prospective randomized comparison study. Clin Orthop Surg.

[21] Riccio M, Battiston B, Pajardi G, Corradi M, Passaretti U, Atzei A (2010). Efficiency of Hyaloglide in the prevention of the recurrence of adhesions after tenolysis of flexor tendons in zone II: a randomized, controlled, multicentre clinical trial. J Hand Surg Eur.

[22] Rothkopf DM, Webb S, Szabo RM, Gelberman RH, May JW (1991). An experimental model for the study of canine flexor tendon adhesions. J Hand Surg Am.

[23] Sanchez Lazaro JA, Granado PC, Del Sol MG, Gonzalez Medina A, Diaz Gallego L, Gonzalez-Arabio Sandoval D (2010). The role of different hyaluronic acids in the articular cartilage of rabbit. Open Orthop J.

[24] Shaffer MA, Okereke E, Esterhai JL, Elliott MA, Walker GA, Yim SH (2000). Effects of immobilization on plantar-flexion torque, fatigue resistance, and functional ability following an ankle fracture. Phys Ther.

[25] Strand V, Baraf HS, Lavin PT, Lim S, Hosokawa H (2012). A multicenter, randomized controlled trial comparing a single intra-articular injection of Gel-200, a new cross-linked formulation of hyaluronic acid, to phosphate buffered saline for treatment of osteoarthritis of the knee. Osteoarthritis Cartilage.

[26] Teeple E, Elsaid KA, Jay GD, Zhang L, Badger GJ, Akelman M, Bliss TF, Fleming BC (2011). Effects of supplemental intra-articular lubricin and hyaluronic acid on the progression of posttraumatic arthritis in the anterior cruciate ligament-deficient rat knee. Am J Sports Med.

[27] Tasto JP, Elias DW (2007). Adhesive capsulitis. Sports Med Arthrosc.

[28] Yan L, Sun Y, Wang J, Dai S, Feng X, Jiang B, Wang Q, Yu T, Shi X, Yang J (2010). The effect of mitomycin C in reducing intraarticular adhesion after knee surgery in rabbits. Eur J Pharmacol.

